# Proceedings of the American Medical Association’s Amended Committee Relative to the Next International Medical Congress

**Published:** 1885-07

**Authors:** 


### EDITORIAL.

PROCEEDINGS OF THE AMERICAN MEDICAL ASSOCIATION'S AMENDED COMMITTEE RELATIVE TO THE MEETING OF THE NEXT INTERNATIONAL MEDICAL CONGRESS.

Pursuant to call, the American Medical Association's Amended Committee on the International Medical Congress met at the Palmer House, in this city, at 12:20 P. M., 24th June, 1885, with the temporary Chairman, Dr. R. BEVERLY COLE, and the temporary Secretary, Dr. JOHN V. SHOEMAKER, in their places.

During the evening session, Dr. A. Y. P. GARNETT, presented an opinion by the Honorable SAMUEL J. RANDALL, ex-Speaker of the United States House of Representatives, relative to the legal status of the committee then in session.

Mr. RANDALL'S opinion indorsed the action of the Association. In a series of able editorials, which have recently appeared in the *Journal of the American Medical Association*, the same view has been taken by the editor of the Association's action at its last annual meeting in New Orleans, as that now expressed by the legal gentleman of the House of Representatives.

The committee met again at the Palmer House on the 25th and received the report of the sub-committee in regard to the Sections into which the Internationl Congress should be divided. They recommended the decrease of the departments to sixteen, and, to accomplish this, grouped several classes of subjects. The report was concurred in. The following were selected as Chairmen of the various classifications of subjects to be discussed: Histology, Professor Stanford E. Chaille, New Orleans; Anatomy, Professor Joseph Leidy, Philadelphia; Physiology, Professor J. C. Dalton, New York; Pathology, Professor Francis Delafied, New York; Practical Medicine, Professor J. M. Da Costa, Philadelphia; Surgery, Professor D. W. Yandell, Louisville; Gynecology, Dr. Robert Battey, Rome, Ga.; Ophthalmology, Professor E. Williams, Cincinnati; Otology, Professor C. J. Blake, Boston; Dermatology, Professor W. O. Hardoway, St. Louis; Laryngology, Professor J. M. McKenzie, Baltimore; Public and International Hygiene, Professor H. A. Johnson, Chicago; Collective Investigation, Nomenclature, Vital Statistics, and Climatology, Professor N. S. Davis, Chicago; Military and Naval Surgery and Medicine, Dr. David L. Huntington, U. S. A.; Practical and Experimental Therapeutics, Professor H. C. Wood, Philadelphia; Diseases of Children Professor J. Lewis Smith, New York. The committee adjourned to meet in St. Louis the first Monday in May, 1866, the day before the annual meeting of the Medical Association. The International Congress will occur in Washington in 1887. The date is not yet fixed, but will probably be some time in August.

It is in the interest of the medical profession of our whole country, and its good name, that selfishness and efforts at personal or partisan advancement should be subordinated, in the work of the Members of the Committee of Arrangements, to the importance of making a success of the next Congress. Their selection by the American Medical Association for this position of trust is no mean honor, but the responsibilities which attach to the position should be appreciated as well, and their course of action should be such as to give undoubted evidence that they realize that fact. No unworthy motive should be allowed to influence the course of any individual member of the committee with any other view than the success of the Congress.

A formal invitation from the medical profession of the United States, by its representatives in the American Medical Association, was extended, through a duly appointed committee of that Association, to the members of the last Congress which met in Copenhagen, to hold the next Congress in the United States. That invitation was accepted. The foreign members of the next Congress will, in their attendance, be in some sense the guests of the medical profession of the United States. The good of the cause, for which the Congress will assemble, should be the governing aim of the committee having charge of the arrangements for it, but this high motive is supplemented by an obligation of hospitality, in addition.

The physicians who will come from foreign countries will neither understand nor sympathize with either side in differences of opinion held by factions in the United States, upon questions of ethics, and still less would they respect any acts of the profession, or of its committee, which might be prompted by selfish or partisan feeling that would conflict with the philanthropic and scientific aim of the Congress.

The character of a large portion of the committee is such that it should be a sufficient guarantee that no unworthy motive would be allowed to characterize the action of the committee as a whole, and that the officers selected shall be representative men, and that the sections formed, to supplement the work of the general sessions of the congress, shall be such as are best suited to advance the cause of scientific medicine.

The medical profession of the United States demands of its representatives—the Committee of Arrangements—that, by no act of theirs, shall the success of the Congress be interfered with nor the good name of the medical profession of our country be made to suffer at their hands, and it looks, hopefully, for just and honorable treatment of the important trust confided to that committee.

